# Nitric Oxide Ameliorates Zinc Oxide Nanoparticles Phytotoxicity in Wheat Seedlings: Implication of the Ascorbate–Glutathione Cycle

**DOI:** 10.3389/fpls.2017.00001

**Published:** 2017-02-06

**Authors:** Durgesh K. Tripathi, Rohit K. Mishra, Swati Singh, Samiksha Singh, Kanchan Vishwakarma, Shivesh Sharma, Vijay P. Singh, Prashant K. Singh, Sheo M. Prasad, Nawal K. Dubey, Avinash C. Pandey, Shivendra Sahi, Devendra K. Chauhan

**Affiliations:** ^1^Centre of Advanced in Botany, Banaras Hindu University VaranasiVaranasi, India; ^2^Centre for Medical Diagnostic and Research, Motilal Nehru National Institute of TechnologyAllahabad, India; ^3^D D Pant Interdisciplinary Research Lab, Department of Botany, University of AllahabadAllahabad, India; ^4^Ranjan Plant Physiology and Biochemistry Laboratory, Department of Botany, University of AllahabadAllahabad, India; ^5^Department of Biotechnology, Motilal Nehru National Institute of TechnologyAllahabad, India; ^6^Government Ramanuj Pratap Singhdev Post Graduate CollegeKoriya, India; ^7^Nanotechnology Application Centre, University of AllahabadAllahabad, India; ^8^Department of Biology, Western Kentucky University, Bowling GreenKY, USA

**Keywords:** amelioration, ascorbate–glutathione cycle, DNA damage, ZnONPs, nanotoxicity, nitric oxide

## Abstract

The present study investigates ameliorative effects of nitric oxide (NO) against zinc oxide nanoparticles (ZnONPs) phytotoxicity in wheat seedlings. ZnONPs exposure hampered growth of wheat seedlings, which coincided with reduced photosynthetic efficiency (F_v_/F_m_ and qP), due to increased accumulation of zinc (Zn) in xylem and phloem saps. However, SNP supplementation partially mitigated the ZnONPs-mediated toxicity through the modulation of photosynthetic activity and Zn accumulation in xylem and phloem saps. Further, the results reveal that ZnONPs treatments enhanced levels of hydrogen peroxide and lipid peroxidation (as malondialdehyde; MDA) due to severely inhibited activities of the following ascorbate–glutatione cycle (AsA–GSH) enzymes: ascorbate peroxidase, glutathione reductase, monodehydroascorbate reductase and dehydroascorbate reductase, and its associated metabolites ascorbate and glutathione. In contrast to this, the addition of SNP together with ZnONPs maintained the cellular functioning of the AsA–GSH cycle properly, hence lesser damage was noticed in comparison to ZnONPs treatments alone. The protective effect of SNP against ZnONPs toxicity on fresh weight (growth) can be reversed by 2-(4carboxy-2-phenyl)-4,4,5,5-tetramethyl- imidazoline-1-oxyl-3-oxide, a NO scavenger, and thus suggesting that NO released from SNP ameliorates ZnONPs toxicity. Overall, the results of the present study have shown the role of NO in the reducing of ZnONPs toxicity through the regulation of accumulation of Zn as well as the functioning of the AsA–GSH cycle.

## Introduction

In recent years, nanotechnology has emerged as a scientific trend that could have benefits in many fields (González-Melendi et al., 2008; Nair et al., 2010; Gogos et al., 2012; Aziz et al., 2015, 2016; Prasad et al., 2016; Tripathi et al., 2017a,c,d). Compared to bulk particles, nano forms show unique physicochemical properties, which are being used as chemical delivery agents in targeting molecules to a specific cellular organelle in plants (Monica and Cremonini, 2009; Nair et al., 2010; Subbaiah et al., 2016; Wang et al., 2016; Tripathi et al., 2017a,b). Several studies have demonstrated both the beneficial and harmful impacts of nanoparticles on plants, which are due to type and size of nanoparticles—especially their specific surface area and the plant species (Moore, 2006; Dietz and Herth, 2011; Miller et al., 2012; Tripathi et al., 2015, 2016; Shweta et al., 2016; Singh et al., 2016). In the ecosystem, plants are not only significant components but also a latent pathway for nanoparticle transport and bioaccumulation into food chains (Zhu et al., 2008; Wang et al., 2012; Zafar et al., 2016).

Among the variety of metal nanoparticles that are frequently used for commercial purposes, ZnONPs are the most prominent. They have been exploited in manufacturing paints, glass, plastics, lubricants, ceramics, pigments, cement, rubber, foods, and batteries (Monica and Cremonini, 2009). Therefore, extensive usage of ZnONPs in various products enhances the probability of their discharge into the environment, which may have serious consequences on plant productivity. Negative impacts of ZnONPs have been tested in various plants like ryegrass, rape, lettuce, radish, corn, cucumber (Lin and Xing, 2007), garden cress and broad bean (Manzo et al., 2011), zucchini (Stampoulis et al., 2009), and wheat (Du et al., 2011). Recent studies also exposed the effect of ZnONPs toxicity on various plant species such as *Cicer arietinum, Brassica nigra, Arabidopsis thaliana, Pisum sativum, Zea mays*, and green alga *Picochlorum* sp. (Burman et al., 2013; Hazeem et al., 2016; Mukherjee et al., 2016; Subbaiah et al., 2016; Wang et al., 2016; Zafar et al., 2016). Thus, the results of previous studies show that ZnONPs have significant negative effects on plant productivity, which demands for developing strategies in order to mitigate ZnONPs toxicity in plants.

Nitric oxide (NO), a gaseous free radical, influences several physiological and biochemical responses in plants under stressful and non-stressful conditions. It has been found that NO effectively alleviates toxic impacts of various stresses like UV-B, salt, heavy metal, heat, and light in plants (Song et al., 2006; Shi et al., 2007; Zhang et al., 2009; Xu et al., 2010; Del Río, 2015; Serrano et al., 2015; Singh et al., 2015). Chen et al. (2015) have reported that NO reduces ZnONPs toxicity in rice seedlings by regulating oxidative damage and antioxidant defense systems. Sufficient literature is available regarding NO-mediated mitigation of metal stress (Saxena and Shekhawat, 2013; Singh et al., 2015; Zhao et al., 2016). However, little is still known about NO-mediated alleviation of nanoparticle toxicity in plants.

In the present study, we have investigated how sodium nitroprusside (SNP; a donor of NO) regulates toxicity of ZnONPs in wheat seedlings by examining: (i) Zn accumulation and physiological and biochemical responses of wheat seedlings under ZnONPs stress, (ii) estimation of enzymes and metabolites of the ascorbate–glutatione cycle (AsA–GSH) cycle under ZnONPs and NO treated seedlings and (iii) mechanisms related to NO-mediated alleviation of ZnONPs stress.

## Materials and Methods

### Synthesis and Characterization of Zinc Oxide Nanoparticles (ZnONPs)

Synthesis of ZnONPs was performed by using the procedure of Sharma et al. (2011) with slight modification. An appropriate amount (21.9 g) of zinc acetate was taken in 100 ml of methanol and dissolved while continuously stirring for 2 h at room temperature. Simultaneously, 140 mM KOH solution was prepared in 100 ml of methanol with refluxing through a water condenser with constant stirring for 2 h at 50°C. Thereafter, both solutions were mixed with constant stirring for 2 h. This mixing was done while refluxing through the water condenser at 50°C. The final solution was allowed to cool at room temperature and agitated overnight. This solution was centrifuged and pellets were washed several times with absolute ethanol and water in order to remove impurities. The obtained product was placed in a vacuum oven for 24 h at 50°C to get powder of ZnONPs. The characterization of ZnONPs was performed by transmission electron microscopy (TEM), UV-Vis spectrophotometer, Raman and X-ray diffraction (XRD) analysis, thermal analysis, and XRD. XRD was performed on a Rigaku D/max-2200 PC diffractometer operated at 40 kV/40 mA, using CuKα1 radiation with wavelength of 1.54 Å in the wide angle region from 20 to 80° on 2𝜃 scale.

### Plant Material and Growth Conditions

Wheat (*Triticum aestivum* L.) seeds were purchased from a certified supplier of local market in the Allahabad district of India. Seeds were surface sterilized in 10% (v/v) sodium hypochlorite solution for 5 min, then washed thoroughly and soaked for 2–4 h in distilled water. After sterilization and soaking, uniform-sized seeds were sown in plastic trays containing sterilized sand. Thereafter, trays were kept in the dark for seed germination at 25 ± 2°C. When germination reached maximum of percentage, seedlings were grown in a growth chamber under photo synthetically active radiation (PAR) of 250 μmol photons m^-2^ s^-1^ and 60% relative humidity with 16:8 h light–dark regime at 25 ± 2°C for 8 days. During the growth period, seedlings were sprayed with water whenever required. Uniformed-sized seedlings were used to analyze the impact of NO on various physiological and biochemical parameters under ZnONPs toxicity.

### Nitric Oxide and ZnONPs Treatments

Uniform-sized seedlings were gently uprooted from sand and their roots washed in tap water. Thereafter, seedlings were acclimatized in half-strength Hoagland’s nutrient solution for 7 days. After this, ZnONPs and NO treatments were given. SNP was used as a donor of NO. Selection of SNP dose (100 μM) was based on earlier studies (Singh et al., 2013, 2015). The treatments include: control (no added ZnONPs and SNP), SNP (100 μM), 100 μM ZnONPs, 100 μM ZnONPs+100 μM SNP, 200 μM ZnONPs and 200 μM ZnONPs+100 μM SNP. In the case of ZnONPs+SNP treatments, seedlings were pretreated with SNP prepared in a nutrient solution for 24 h under PAR of 250 μmol photons m^-2^ s^-1^. They were then given ZnONPs treatments. Just after ZnONPs and SNP treatments, seedlings were further grown in a growth chamber for 7 days under 250 μmol photons m^-2^ s^-1^ of PAR and 60% relative humidity with 16:8 h light–dark regime at 25 ± 2°C. During this growth period, the medium of various treatments was changed twice and aerated daily to avoid root anoxia. After 7 days of ZnONPs and SNP treatments, seedlings were harvested and various parameters were analyzed immediately. To test whether NO released from SNP is involved in ZnONPs toxicity alleviation, 2-(4-carboxy-2-phenyl)-4, 4, 5, 5-tetramethylimidazoline-1-oxyl-3-oxide (c-PTIO, 100 μM), a scavenger of NO, was used.

### Estimation of Growth, Photosynthetic Pigments, Chlorophyll Fluorescence, and NO Content

Growth was measured in terms of fresh weight. Seedlings were selected randomly from control and treated samples and then their fresh weight was determined. For the estimation of photosynthetic pigments (total chlorophyll, chlorophyll *a* + chlorophyll *b*), the method of Lichtenthaler (1987) was adopted. For the assessment of photosynthetic performance, chlorophyll *a* fluorescence measurements were taken in the dark adapted leaves of control and treated seedlings using hand held leaf fluorometer (FluorPen FP 100, Photos System Instrument, Czech Republic). The estimation of NO was performed according to the method of Zhou et al. (2005) as described in Singh et al. (2015).

### Estimation of Zn Content in Seedlings

For the determination of Zn content, dried samples (50 mg) from control and treated seedlings were digested in mixed acid (HNO_3_:HClO_4_; 85:15, v/v) until transparent solution was obtained. The volume of the digested sample was maintained up to 30 ml with double distilled water. The content of Zn in digested samples was determined by an atomic absorption spectrometer (iCE 3000 Series, model-3500 AAS, Thermo scientific, UK) fitted with a specific lamp of particular metal using the appropriate drift blank.

### Collection of Phloem and Xylem Saps, and Estimation of Zn Content

The collection of phloem and xylem saps from wheat seedlings was performed according to the method of Hazama et al. (2015). Treated and untreated seedlings of 27 days cut on surfaces of the stems near the petioles of mature leaves with a razor blade and exuded drops (excluding the first drop) collected as phloem sap using micropipettes. Samples of phloem sap were stored in eppendorf previously washed with 0.1 M HNO_3_ for 2 days and then with double distilled water three times to eliminate metals. The samples were stored at -20°C until analysis.

After the collection of phloem sap, stems were cut at 2 cm above the interface of the shoot and root, and xylem sap exudates were collected for 30 min using micropipettes. The measured pH of the xylem sap was 6.0–6.4. Samples of xylem sap were also stored at -20°C until analysis.

For the estimation of Zn in phloem and xylem saps above, the described procedure (for Zn) was followed.

### Estimation of Hydrogen Peroxide (H_2_O_2_) and Lipid Peroxidation

For the estimation of H_2_O_2_, fresh samples (50 mg) from control and treated seedlings were crushed in 0.1% (w/v) trichloroacetic acid (Velikova et al., 2000). Absorbance of the reaction mixture was recorded at 390 nm. The H_2_O_2_ concentration was calculated by using a standard curve prepared with H_2_O_2_. Lipid peroxidation as MDA content was estimated according to the method of Heath and Packer (1968). The values of non-specific absorption recorded at 600 nm were subtracted from the values recorded at 532 nm. The content of MDA was determined by using an extinction coefficient of 155 mM^-1^ cm^-1^.

### Estimation of Activities of APX, GR, MDHAR, and DHAR: the Ascorbate–Glutathione Cycle Enzymes

Fresh samples (1.0 g) from control and treated seedlings were homogenized in 10 ml of chilled 50 mM potassium phosphate buffer (pH 7.0) containing 1 mM EDTA and 1% (w/v) polyvinylpyrrolidone in mortar and pestle under cool conditions. In the case of APX and DHAR activities, 1 mM ascorbic acid and 2 mM 2-mercaptoethanol were added into the above buffer, respectively. The homogenate was centrifuged at 20,000 *g* for 10 min at 4°C and supernatant was used as an enzyme. All enzymatic measurements were carried out at 25°C by using a Shimadzu, UV-VIS Spectrophotometer (UV-1700 Pharma Spec) (Gangwar et al., 2011).

APX (EC 1.11.1.11) activity was determined according to the method of Nakano and Asada (1981). The decrease in absorbance was measured at 290 nm. The enzyme activity was calculated by using an extinction coefficient of 2.8 mM^-1^ cm^-1^. One unit (U) of enzyme activity is defined as 1 nmol ascorbate oxidized min^-1^.

Glutathione reductase (EC 1.6.4.2) activity was assayed according to the method of Schaedle and Bassham (1977). The decrease in absorbance was read at 340 nm, and GR activity was calculated using an extinction coefficient of 6.2 mM^-1^ cm^-1^. One unit (U) of enzyme activity is defined as 1 nmol NADPH oxidized min^-1^.

Monodehydroascorbate reductase (EC 1.6.5.4) activity was estimated according to the method of Hossain et al. (1984). The enzyme activity was calculated using an extinction coefficient of 6.2 mM^-1^ cm^-1^. One unit (U) of enzyme activity is defined as nmol NADPH oxidized min^-1^.

Dehydroascorbate reductase (EC 1.8.5.1) activity was assayed by the method of Nakano and Asada (1981). An increase in absorbance was read at 265 nm, and DHAR activity was calculated using an extinction coefficient of 7.0 mM^-1^ cm^-1^. One unit (U) of enzyme activity is defined as 1 nmol DHA reduced min^-1^.

### Estimation of Metabolites of the Ascorbate–Glutathione Cycle: Ascorbate and Glutathione

Total ascorbate, AsA, and DHA were determined by the method of Gossett et al. (1994). This assay is based on the reduction of Fe^3+^ into Fe^2+^ with ascorbic acid in acid solution followed by formation of red chelate between Fe^2+^ and 2, 2′- bipyridyl. Control and treated samples were homogenized in 10 ml of 5% (w/v) m-phosphoric acid using a mortar and pestle in cool conditions. The homogenate was centrifuged at 22,000 *g* for 15 min. AsA+DHA was determined in a reaction mixture consisting of 0.2 ml of supernatant, 0.5 ml of a 150 mM potassium phosphate buffer (pH 7.4) containing 5 mM EDTA and 0.1 ml of 10 mM DTT to reduce DHA to AsA. After 10 min of incubation at room temperature, 0.1 ml of 0.5% (w/v) *N*-ethylmaleimide was mixed in samples and stirred for 5 min to remove excess DTT. AsA was assayed in a similar manner, except that DTT was substituted by 200 μl deionized H_2_O. Color developed in the reaction mixture through the addition of 0.4 ml of 10% (w/v) TCA, 0.4 ml of 44% (v/v) o-phosphoric acid, 0.4 ml of 4% (w/v) 2, 2′-bipyridyl in 70% (v/v) ethanol and 0.2 ml of 3% (w/v) FeCl_3_. The reaction mixture was incubated at 40°C for 1 h and quantified spectrophotometrically at 525 nm. DHA was determined by subtracting AsA from AsA+DHA. Ascorbate content was found out by using a standard curve prepared with L-ascorbic acid.

Total (GSH + GSSG), reduced (GSH), and GSSG contents were estimated using the enzyme recycling method of Brehe and Burch (1976). This method is based on the sequential oxidation of GSH by 5, 5-dithiobis-2-nitrobenzoic acid (DTNB) and the reduction of GSSG in the presence of NADPH and GR (type III from bakers’ yeast; Sigma Chemical Company). Control and treated samples were crushed in 3 ml of 6% (w/v) *m*-phosphoric acid containing 1 mM EDTA using a mortar and pestle and centrifuged at 1000 *g* for 10 min. The reaction mixture (2.4 ml) contained 0.8 ml of reagent A [110 mM Na_2_HPO_4._ 7H_2_O, 40 mM NaH_2_PO_4_. H_2_O, 15 mM EDTA, 0.3 mM DTNB and 0.04% (w/v) BSA], 0.64 ml reagent B [1 mM EDTA, 50 mM imidazole and 0.02% (w/v) BSA], which contained an equivalent of 1.5 units GR activity ml^-1^ and 0.8 ml of a 1:20 dilution of acid extract in 5% (w/v) Na_2_HPO_4_ (pH 7.5). The dilution of acid extract was done immediately prior to starting the reaction through the addition of 0.16 ml of 3 mM NADPH. The change in absorbance of the reaction mixture was measured at 412 nm for 5 min. GSSG was estimated by first incubating 1 ml of 1:20 diluted extract with 40 μl of 2-vinylpyridine for 1 h at 25°C with vigorous shaking. The samples incubated with 2-vinylpyridine were used for the assay of GSSG content. The amount of GSH was determined by subtracting GSSG from GSH+GSSG using a standard curve prepared with GSH.

### Statistical Analysis

Results were statistically analyzed by an analysis of variance (ANOVA). Duncan’s multiple range test was applied for mean separation for significant differences among treatments at the *P* < 0.05 significance level. The results presented are the mean ± standard error of six replicates (*n* = 6).

## Results

### Observation of ZnONPs

The UV-Vis spectrum of prepared ZnONPs solution is shown in Supplementary Figure S1A. The prepared ZnONPs have a blue-shifted absorbance peak approximately at 340 nm compared with that of the bulk ZnO, which has an absorbance peak at 370 nm corresponding to a 3.35 eV band gap at room temperature. Supplementary Figure S1B shows typical Raman-scattering spectrum of the ZnONPs performed at room temperature. A sharp, strong, and dominant peak located at about 438 cm^-1^ was observed, which was characteristic of the scattering peak of the Raman active dominant E_2_ (high) mode of wurtzite hexagonal ZnONPs. In addition, some very weak peaks at 331 cm^-1^ were also observed, which were assigned to E_2H_–E_2L_ (multi-phonon). Some broadened and weak peaks at 580 cm^-1^ also appeared in the spectrum.

The available reflections of the present XRD phases have been fitted with Gaussian distribution. The broadening of XRD peaks (i.e., Scherrer’s broadening) attributes the formation of ZnONPs. The crystallite size, d, of ZnONPs was estimated by Debye–Scherrer’s equation:

$$d=\frac{0.9\lambda }{\beta \;Cos\theta }$$

where, d is the crystallite size, λ is the wavelength of radiation used, θ is the Bragg angle, and θ is the full width at half maxima (FWHM) on 2θ scale. The crystallite size was estimated for the most prominent X-ray diffraction, corresponding to a peak at 36.25 °, and was deduced to be 15–18 nm (Supplementary Figure S1C).

The variations in XRD results were well-supported by TEM measurements. Supplementary Figure S1D shows the representative TEM image of the prepared sample. The morphology of the sample was found to be nearly spherical in nature having diameters ranging from 5 to 20 nm. Supplementary Figure S1E shows the particle size distribution with the mean at 15.37 nm.

Supplementary Figure S1F shows that thermal analysis of ZnONPs. The ZnONPs had a very small weight loss (-2.5 wt %) below 300 ° C, probably due to the removal of physically and chemically adsorbed water on their surfaces. The second weight loss (-15 wt%) at about 300 ° C is assigned to the decomposition of remaining Zn(OH)_2_.

### Growth and NO and Zn Accumulation

**FIGURE 1:**
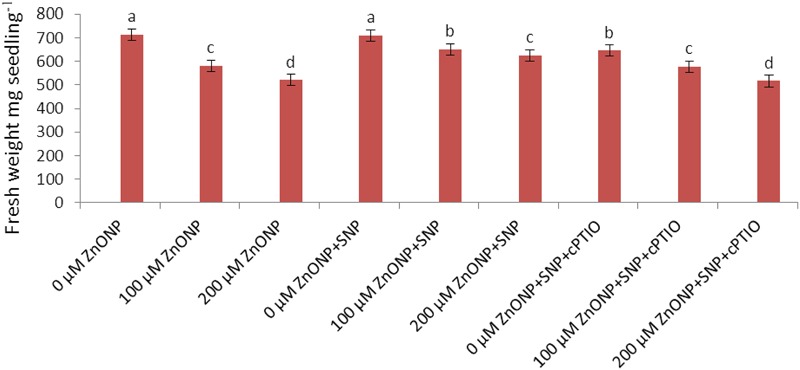
**Impact of SNP and cPTIO interaction on alleviation of ZnONPs toxicity.** Data are mean ± standard error of six replicates (*n* = 6). Bars followed by different letter(s) show a significant difference at the *P* < 0.05 significance level according to the Duncan’s multiple ranges test.

Growth was measured in terms of fresh weight and declined significantly (*P* < 0.05) following ZnONPs treatments (**Figure 1**). Treatment of the wheat seedling with 100 and 200 μM ZnONPs resulted in a significant decline in fresh weight by 19 and 28% respectively as compared to the control. The addition of NO donor, i.e., SNP significantly (*P* < 0.05) alleviated the ZnONPs-induced decline and showed a decrease of only 9 and 17% in fresh weight respectively over the value of the control (**Figure 1**). However, the addition of c-PTIO, a scavenger of NO, reverses the NO-mediated alleviative effect on ZnONPs toxicity (**Figure 1**), suggesting a role for SNP released NO in ameliorating ZnONPs toxicity.

Wheat seedlings grown under 100 and 200 μM ZnONPs treatments accumulated about 391.4 ± 3.9 and 542.3 ± 5.4 μg Zn g^-1^ dry weight. On the other hand, the addition of SNP (100 μM ZnONPs + SNP and 200 μM ZnONPs +SNP) significantly lowered the excess enhancement of ZnONPs in plants. Accumulation was only187.2 ± 1.8 and 289.5 ± 2.9 μg Zn g^-1^ dry weight (**Table 1**).

**Table 1:** Effect of SNP on NO and Zn contents in seedlings and xylem and phloem saps exposed to ZnONPs phytotoxicity.

Treatments	NO content (ng g^-1^ dry weight)	Zn in seedlings (mg kg^-1^ dry)	Zn content (ng ml^-1^)	
			Phloem sap	xylem sap
**ZnONP (μM)**				
0	76.5 ± 2.6d	92.6 ± 2.6f	2011.2 ± 24.1e	242.3 ± 3.8e
100	55.8 ± 1.4e	391.4 ± 3.9b	4484.9 ± 27.2b	566.9 ± 4.6b
200	41.8 ± 1.3f	542.3 ± 5.4a	6395.6 ± 32.4a	811.7 ± 5.3a
**ZnONP (μM)+SNP(μM)**				
0	96.7 ± 3.8b	98.6 ± 3.5e	2005 ± 19.6e	245.3 ± 3.6e
100	88.9 ± 2.7c	187.2 ± 1.8d	3077.1 ± 24.5d	346.5 ± 3.8d
200	127.8 ± 3.1a	289.5 ± 2.9c	3881.6 ± 27.3c	513.8 ± 4.2c

*Data are mean ± standard error of six replicates (*n* = 6). The values followed by different letters within the same column are significantly different at *P* < 0.05 significance level according to the Duncan’s multiple ranges test.*

Similar to the results of growth, the exposure of 100 and 200 μM ZnONPs decreased NO content by 27 and 45%, respectively compared to the control (**Table 1**). The addition of SNP together with both doses of ZnONPs significantly raised levels of NO in wheat seedlings compared to the even control sample (**Table 1**).

### Impact of NO on Photosynthetic Pigments and Chlorophyll A Fluorescence under ZnONPs Phytotoxicity

Total chlorophyll content decreased by 17 and 28% exposed to 100 and 200 μM of ZnONPs respectively over the value of control. However, the addition of SNP significantly alleviates the adverse impact, and reductions were only 6 and 12% under combinations of SNP+100 μM ZnONPs and SNP+200 μM ZnONPs respectively. SNP alone showed significant improvement in chlorophyll content (**Table 2**).

**Table 2:** Effects of SNP on total chlorophyll (total Chl), maximum photochemical efficiency of PS II (F_v_/F_m_), qP and NPQ in wheat seedlings exposed to exposed to ZnONPs phytotoxicity.

Treatments	Total chlorophyll(mg g^-1^ fresh weight)	F_v_/F_m_	qP	NPQ
**ZnONP (μM)**				
0	1.74 ± 0.028ab	0.855 ± 0.009bc	0.760 ± 0.011a	1.34 ± 0.012f
100	1.45 ± 0.024d	0.786 ± 0.009e	0.675 ± 0.010bc	1.98 ± 0.017b
200	1.25 ± 0.020de	0.653 ± 0.007f	0.523 ± 0.008d	2.43 ± 0.021a
**ZnO NP (μM)+SNP (100 μM)**		
0	1.88 ± 0.031a	0.981 ± 0.011a	0.753 ± 0.011a	1.45 ± 0.013e
100	1.63 ± 0.027b	0.849 ± 0.010b	0.714 ± 0.010b	1.63 ± 0.014d
200	1.53 ± 0.025bc	0.839 ± 0.010cd	0.689 ± 0.010bc	1.87 ± 0.016bc

*Data are means ± standard error of six replicates (*n* = 6). Values followed by different letters within the same column are significantly different at *P* < 0.05 significance level according to the Duncan’s multiple ranges test.*

The results pertaining to chlorophyll fluorescence showed that the exposure of ZnONPs (100 and 200 μM) significantly declined F_v_/F_m_ by 8 and 24% and qP by 11 and 31%compared to their respective controls (**Table 2**). In contrast, the addition of NO successfully alleviated the ZnONPs induced decline in F_v_/F_m_ and qP as reductions were only 1 and 2% in F_v_/F_m_ and 6 and 9% in qP respectively. Moreover, the addition of NO alone significantly improved the levels of F_v_/F_m_ and qP when compared to the respective controls. NPQ was enhanced by ZnONPs treatments by 48 and 81%, respectively. However, NO addition along with both the ZnONPs treatments significantly (*P* < 0.05) reduced the ZnONps-mediated increments in NPQ (**Table 2**).

### Concentrations of Zn in Xylem and Phloem Saps

Nitric oxide treatment alone does not show any remarkable change in the concentration of Zn in xylem sap (**Table 1**). But under both the concentration of ZnONps, concentrations of Zn in xylem sap were enhanced by 134 and 235% as compared to the respective control. On the other hand, SNP addition along with both the doses of ZnONPs lowered the enhanced concentrations of Zn in xylem sap, which were only 43 and 112% as compared to their respective controls (**Table 1**).

Under treatments of ZnONPs (100 and 200 μM), concentrations of Zn in the phloem sap were enhanced by 123 and 218% as compared to the respective control (**Table 1**). In contrast, SNP addition (100 μM ZnONPs + SNP and 200 μM ZnONPs +SNP) drastically lowered Zn concentrations in the phloem sap, which were only 53 and 93% as compared to ZnONPs treatments alone (**Table 1**).

### Impact of NO on H_2_O_2_ and Lipid Peroxidation under ZnONPs Phytotoxicity

**FIGURE 2:**
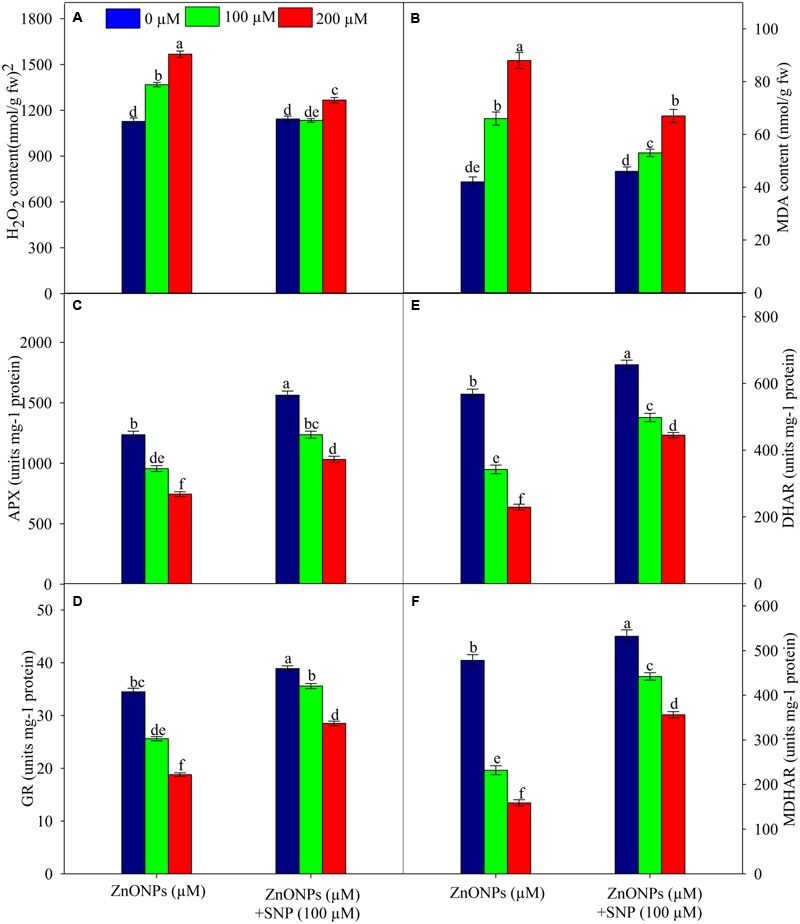
**Effects of SNP on H_2_O_2_ (A)**, lipid peroxidation **(B)**, activity of APX **(C)**, GR **(D)** DHAR **(E)** and MDHAR **(F)** in wheat seedlings exposed to ZnONPs phytotoxicity. Data are means ± standard error of six replicates (*n* = 6). Bars followed by different letter(s) show a significant difference at *P* < 0.05 significance level according to the Duncan’s multiple ranges test.

The results related to the oxidative stress markers reveal that 100 and 200 μM of ZnONPs raise H_2_O_2_ and MDA contents by 21 and 39% and 57 and 109%, respectively as compared to the value of respective control (**Figures 2A,B**). In contrast to ZnONPs treatments, the addition of SNP along with ZnONPs significantly (*P* < 0.05) lowered the ZnONPs-mediated enhancement of H_2_O_2_ and MDA contents in wheat seedlings as their contents were increased only by 7 and 12% and 26 and 59%, respectively in 100 μM ZnONPs+SNP and 200 μM ZnONPs+SNP treated seedlings (**Figures 2A,B**). Furthermore, when SNP was added alone in wheat seedlings, it does not show any significant (*P* < 0.05) alteration in H_2_O_2_ and MDA contents as compared to the value of the respective control (**Figures 2A,B**).

### Impact of NO on the Activities of Enzymes of the Ascorbate–Glutathione Cycle under ZnONPs Phytotoxicity

The results related to the activities of the AsA–GSH cycle enzymes showed that under ZnONPs treatments (100 and 200 μM) APX, GR, DHAR, and MDHAR were significantly inhibited. For instance, both ZnONPs treatments declined (*P* < 0.05) APX activity by 22 and 39%, GR activity by 25 and 45%, DHAR activity by 39 and 59%, and MDHAR by 51 and 66%, respectively (**Figures 2C–F**). However, SNP treatment alone significantly (*P* < 0.05) enhanced the activities of all enzymes studied. It was also observed that due to the addition of SNP along with ZnONPs (100 μM ZnONPs+SNP and 200 μM ZnONPs+SNP), activities of APX, GR, DHAR, and MDHAR were significantly improved when compared to ZnONPs treated wheat seedlings (**Figures 2C–F**). This shows the positive impact of SNP against the adverse impact of ZnONPs on the AsA–GSH cycle enzymes in wheat seedlings.

### Impact of NO on Ascorbate and Glutathione Contents under ZnONPs Stress

The results related to ASA and DHA contents of wheat seedlings treated with and without ZnONPs (100 and 200 μM) are depicted in **Table 3**. The results showed that the AsA+DHA and AsA were significantly (*P* < 0.05) enhanced under both the ZnONPs treatments. Yet, the ASA ascorbate/dehydroascorbate ratio severely declined under ZnONPs treatments. The addition of SNP together with ZnONPs (100 μM ZnONPs+SNP and 200 μM ZnONPs+SNP) stimulated the ratio of AsA/DHA as compared to ZnONPs alone (**Table 3**).

**Table 3:** Effects of SNP on AsA+DHA, AsA, DHA and AsA/DHA, GSH+GSSG, GSH and GSH/GSSG in wheat seedlings exposed to ZnONPs phytotoxicity.

Treatments	Total ascorbate and glutathione (nmol g^-1^ fresh weight)							
**ZnO NP (μM)**	**AsA+DHA**	**AsA**	**DHA**	**AsA/DHA**	**GSH + GSSG**	**GSH**	**GSSG**	**GSH/GSSG**

0	464.4 ± 5.9ef	429.5 ± 4.7e	34.9 ± 0.46de	12.3 ± 0.07a	716.8 ± 6.2f	670.8 ± 9.6e	45.9 ± 0.31ef	14.6 ± 0.14a
100	488.3 ± 6.2cd	442.3 ± 4.9c	46.1 ± 0.61c	9.6 ± 0.07c	766.5 ± 6.7d	695.5 ± 9.6cd	70.9 ± 0.52b	9.8 ± 0.09d
200	517.8 ± 6.5b	440.5 ± 4.8c	77.3 ± 1.0a	5.7 ± 0.03d	807.2 ± 7.0b	702.4 ± 10.0c	104.8 ± 0.71a	6.7 ± 0.07e
**ZnO NP (μM)+SNP (100 μM)**							
0	470.9 ± 5.9de	436.3 ± 4.8cd	34.6 ± 0.45de	12.6 ± 0.07a	745.6 ± 6.5e	699.6 ± 10.0cd	46 ± 0.31e	15.2 ± 0.15a
100	497.8 ± 6.3bc	458.9 ± 5.1b	38.8 ± 0.51d	11.8 ± 0.07ab	788.5 ± 6.9c	734.1 ± 10.5b	54.4 ± 0.37d	13.5 ± 0.13b
200	541.5 ± 6.8a	490.4 ± 5.4a	51.1 ± 0.68b	59.6 ± 0.06c	893.1 ± 7.8a	826.9 ± 11.8a	66.2 ± 0.45bc	12.5 ± 0.12bc

*Data are means ± standard error of six replicates (*n* = 6). Values followed by different letters within the same column are significantly different at *P* < 0.05 significance level according to the Duncan’s multiple ranges test.*

The contents of glutathione were also estimated in wheat seedlings treated with ZnONPs. The results suggested that GSH + GSSG, GSH, and GSSG were enhanced under ZnONPs (100 and 200 μM) as GSH + GSSG increased by 6 and 12%, GSH contents by 3 and 4%, and GSSG by 45 and 128%, respectively. In contrast, the content of GSH/GSSG significantly declined under both the ZnONPs treatments (**Table 3**). Interestingly, SNP together with ZnONPs treatments stimulated concentrations of studied glutathione species and ratios of GSH/GSSG (**Table 3**).

## Discussion

In the present study, ZnONPs toxicity was observed in wheat seedlings exposed to 100 and 200 μM of ZnONPs (**Figure 1**; **Table 1**). The results show that ZnONPs caused alterations in photosynthetic pigments, the level of antioxidants, and photosynthetic performance (F_v_/F_m_ and qP). It also enhanced levels of oxidative stress markers such as H_2_O_2_ and MDA. These alterations may be related to reduced growth of wheat seedlings under ZnONPs toxicity (**Tables 1–3**; **Figure 1**). Similar to our results, recent studies also show ZnONPs-mediated toxicity in several plants species (Burman et al., 2013; Hazeem et al., 2016; Mukherjee et al., 2016; Subbaiah et al., 2016; Wang et al., 2016; Zafar et al., 2016). Considering mechanisms of nanoparticle toxicity in plants, several modes of toxicity have been proposed (Brunner et al., 2006; Subbaiah et al., 2016). In the present study with regard to toxic action of ZnONPs in plants, it can be stated that ZnONps exposure caused enhanced generation of ROS and altered antioxidant defense systems, which resulted in damage to crucial macromolecules such as lipids and hence hindered growth (**Table 1**; **Figures 1** and **2**).

However, the addition of SNP (a donor of NO) alleviated ZnONPs-induced phytotoxicity in wheat seedlings (**Figure 1**; **Table 1**). Chen et al. (2015) have shown that NO mitigates ZnONPs-induced phytotoxicity in rice seedlings by regulating oxidative stress and antioxidant defense systems. Further, Singh et al. (2016) reported that NO mitigates As^V^ toxicity in rice seedlings by regulating As accumulation and antioxidant defense system. Our results show that NO supplementation regulates the accumulation of Zn, ROS, and lipid peroxidation (MDA) (**Tables 1** and **3**; **Figures 1** and **2**). NO-mediated lowering in the accumulation of Zn under ZnONPs phytotoxicity may be one of the reasons of mitigation of ZnONPs phytotoxicity in wheat seedlings. In the case of NO-mediated mitigation of metal toxicity, there are three proposed mechanisms: (1) direct neutralization of metal induced-ROS and enhancement in antioxidant contents/activity (Singh et al., 2016) (2) alteration in components of cell walls that ultimately governs the uptake of metals (Singh et al., 2013, 2015), and (3) the signaling of NO molecule that leads changes in the expression of a set of stress responsive genes (Shaibur and Kawai, 2009; Xu et al., 2010; Chen et al., 2015; Singh et al., 2016).

Under stressed conditions, the fine regulation of ROS is essential for the cellular homeostasis and survival of plants. In the cell, the balance of ROS is maintained by enzymatic as well as non-enzymatic antioxidants. The AsA–GSH cycle is a major H_2_O_2_ scavenging pathway that operates both in the chloroplast and cytosol and contains some antioxidant enzymes, i.e., APX, MDHAR, GR, and DHAR and two non-enzymatic antioxidants, i.e., ascorbate and glutathione (Foyer and Noctor, 2011; Singh et al., 2015). The results show that ZnONPs (*P* < 0.05) inhibited activities of APX, GR, MDHAR, and DHAR, which resulted into reduced ratios of AsA/DHA and GSH/GSSG (**Figures 2C–F**; **Table 3**). Inhibitions in GR and MDHAR and DHAR activities coincided with an increased (*P* < 0.05) pool of GSSG and DHA respectively (**Table 3**; **Figures 2C–F**). This condition leads to a buildup of H_2_O_2_ and oxidative damage because APX reduces H_2_O_2_ into H_2_O and O_2_ using AsA. It is known that GSH and AsA are crucial non-enzymatic antioxidants. They stabilize the structure of the membrane within the cell. In addition to this °OH, the most hazardous form of ROS is scavenged by AsA, while GSH is used to render toxic cellular products into non-toxic ones for which no enzymatic system has evolved (Foyer and Noctor, 2011). In contrast, the addition of SNP together with ZnONPs triggers up-regulation of activities of the AsA–GSH cycle enzymes and amounts of its associated metabolites-AsA and GSH. Therefore, under ZnONPs stress, up-regulation of the AsA–GSH cycle due to SNP addition may be the second reason for the alleviation of ZnONPs phytotoxicity in wheat seedlings.

## Conclusion

The results of the present study show that there may two ways through which NO regulates ZnONPs toxicity in wheat seedlings. (1) NO decreased the accumulation of excess Zn in xylem and phloem saps, which resulted in lesser generation of oxidative stress as indicated by lower amounts of MDA. (2) NO triggers up-regulation of the AsA–GSH cycle enzymes and its associated metabolites, which provide better protection to wheat seedlings against ZnONPs-mediated oxidative stress. Results show that the addition of c-PTIO reverses the SNP-mediated alleviatory effect, suggesting a role of SNP released NO in ameliorating ZnONPs toxicity in wheat seedlings. Overall, the results suggest the involvement of NO in the reduction of ZnONPs toxicity in wheat seedlings. These results may be helpful in protecting wheat crops in particular and other plants in general against the effects of nanotoxicity.

## Author Contributions

DT, VS, DC, SP, and ND designed experiments. DT, RM, SwS, KV, and PS performed experiments. DT, RM, SaS, SSh, VS, PS, DC, SP,ND, SSa, and AP analyzed data and wrote the manuscript.

## Conflict of Interest Statement

The authors declare that the research was conducted in the absence of any commercial or financial relationships that could be construed as a potential conflict of interest.

## Abbreviations

APX: ascorbate peroxidase
AsA: reduced ascorbate
AsA+DHA: total ascorbate
DHA: dehydroascorbate
DHAR: dehydroascorbate reductase
F_v_/F_m_: maximum photochemical efficiency of PS II
GR: glutathione reductase
GSH: reduced glutathione
GSH+GSSG: total glutathione
GSSG: oxidized glutathione
H_2_O_2_: hydrogen peroxide
MDA: malondialdehyde
MDHAR: monodehydroascorbate reductase
NPQ: non-photochemical quenching
qP: photochemical quenching
ROS: reactive oxygen species
ZnONPs: zinc oxide nanoparticles
