# Supplementary material for: Nitric Oxide Ameliorates Zinc Oxide Nanoparticles Phytotoxicity in Wheat Seedlings: Implication of the Ascorbate–Glutathione Cycle
Source: Front Plant Sci. 2017 Feb 6;8:1. doi: 10.3389/fpls.2017.00001 (PMC5292406; doi:10.3389/fpls.2017.00001)
Supplement: Supplementary file 1 [file Data_Sheet_1.docx]

**Supplementary material:**

**Fig.S1.** Characterization of ZnO nanoparticles (ZnONPs) by various techniques. The UV-Vis absorption spectrum of synthesized ZnONPs (A), Raman-scattering spectrum of the ZnONPs (B), XRD pattern of synthesized ZnONPs (C), TEM micrograph of ZnONPs (D), histogram of particle size (E) and thermal analysis of synthesized ZnONPs (F)


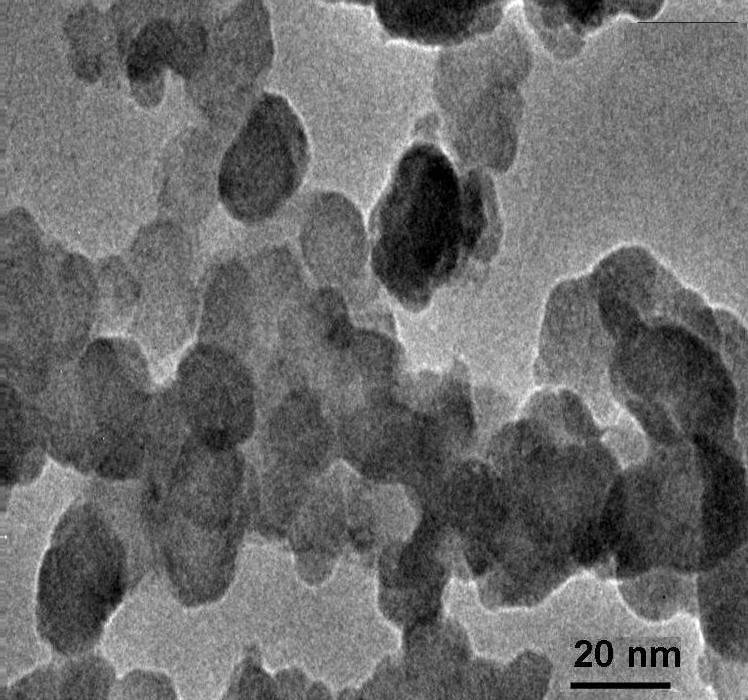

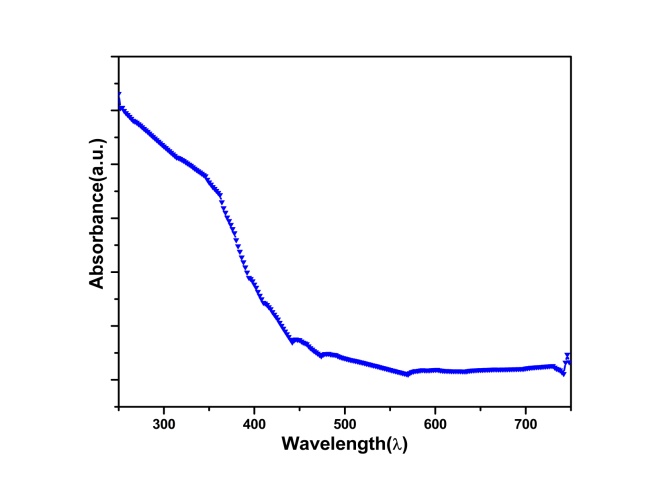

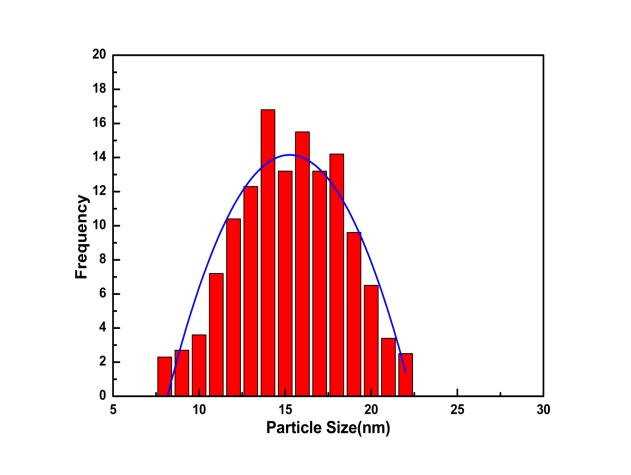

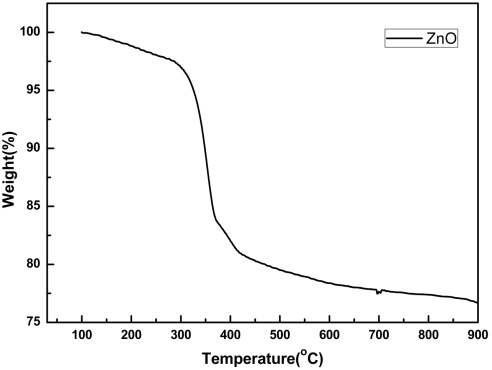

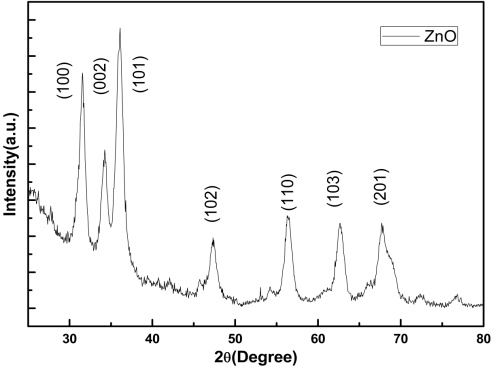


(A)

(D)

(E)

(B)

(F)

(C)

(A)

**Fig.S1**
